# Comparison of Two l‑Arabinose Isomerases for Multienzymatic Conversion of Lactose in Skim Milk Permeate at Neutral and Acidic pH

**DOI:** 10.1021/acs.jafc.5c04545

**Published:** 2025-06-12

**Authors:** Nathanael Weber, Sabine Lutz-Wahl, Lutz Fischer

**Affiliations:** Institute of Food Science and Biotechnology, Department of Biotechnology and Enzyme Science, 26558 University of Hohenheim, Garbenstr. 25, 70599 Stuttgart, Germany

**Keywords:** d-tagatose, l-arabinose isomerase, β-galactosidase, xylose isomerase, d-galactose isomerization, lactose hydrolysis, glucose isomerization, skim milk ultrafiltration permeate

## Abstract

The sweetness of dairy byproducts can be increased by hydrolysis of lactose to d-glucose and d-galactose and subsequent isomerization to d-fructose and d-tagatose. The isomerization of d-galactose to d-tagatose in skim milk ultrafiltration permeate at pH 4.5 and 6.5 was investigated here, comparing the l-arabinose isomerase from Lentilactobacillus parakefiri (l-AI-Lp) with l-AI-N13, a protein-engineered variant of l-AI from Geobacillus stearothermophilus. l-AI-Lp combined with a commercial β-galactosidase and xylose isomerase converted 95 g/L of lactose to d-glucose, d-galactose, d-fructose, and d-tagatose (23–24 g/L each) at pH 6.5. At pH 4.5, lactose (100 g/L) was converted by a commercial β-galactosidase and l-AI-Lp to 46 g/L d-glucose, 22 g/L d-galactose, and 23 g/L d-tagatose, while no activity was measured for l-AI-N13 at this pH. This is the first study that demonstrates the enzymatic conversion of lactose in a dairy byproduct to d-glucose, d-galactose, and d-tagatose at an acidic pH.

## Graphical abstract

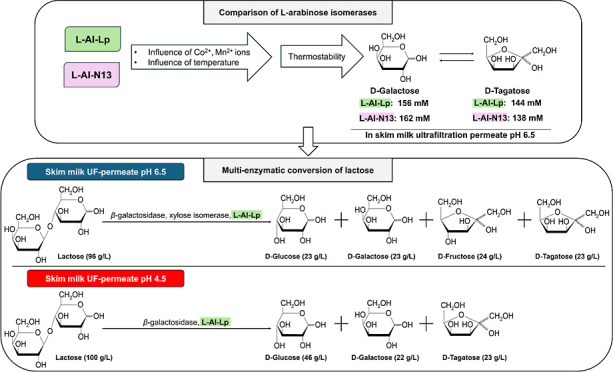

## 1 Introduction

In 2023, approximately 155.1 million tons of raw bovine milk were produced in the European Union, including 31.5 million tons in Germany.^1^ This milk was subsequently processed into various dairy products, such as 2,656,881 tons of cheese and 521,456 tons of cream in Germany that same year.^2^ The production of cream generates skim milk as a byproduct, which can be further processed using ultrafiltration to concentrate milk proteins in the retentate fraction, yielding skim milk ultrafiltration permeate (skim milk UF-permeate).^3^ Another byproduct of the dairy industry is sweet whey, which is produced at a ratio of approximately 9 kg per kg of cheese manufactured.^4^ Research has been done on the valorization of skim milk UF-permeate and sweet whey, focusing on the production of galactooligosaccharides and organic acids, as well as the recovery of whey proteins.^5–8^ By contrast, the utilization of acid whey, which is a byproduct of the production of Greek-style yogurt and acid-coagulated cheese, is still difficult due to its lower pH (pH 4.2–4.6) and higher calcium content (approximately 0.7–1.3 g/L).^9^

Furthermore, other studies have investigated the multienzymatic conversion of lactose in UF-permeates of skim milk and sweet and acid whey to increase their sweetness and expand their potential applications as sucrose substitutes.^10,11^ Lactose, which has a relative sweetness (RS) of only 16 compared to sucrose (RS = 100), was first hydrolyzed by β-galactosidase (β-gal; EC 3.2.1.23) to give d-glucose (RS = 74) and d-galactose (RS = 32).^10,11^ The sweetness was further increased by isomerizing d-glucose to d-fructose (RS = 173) using a xylose isomerase (XI; EC 5.3.1.5).^10,11^ Luzzi et al. applied this bienzymatic method in the production of yoghurt and pudding, resulting in a reduction of 10%–20% (w/w) in the total sugar content while maintaining the same sweetness.^10^ Further studies extended the process by isomerizing d-galactose to d-tagatose (RS = 92) using an l-arabinose isomerase (l-AI; EC 5.3.1.4).^12,13^ Shen et al. demonstrated a 440% increase in the RS of cheese whey permeate by applying this trienzymatic process.^12^ However, all multienzymatic conversions have been limited to neutral pH conditions due to the low activity and stability of the applied enzymes at acidic pH.^10–13^

d-Tagatose is a naturally occurring sweetener that can be used as a substitute for sucrose in food products due to its similar sweetness, lower physiological caloric value of 12.5 kJ/g (sucrose = 17 kJ/g), and similar sensory characteristics.^14–16^ In addition, it has been authorized as a novel food ingredient in the European Union since 2005.^16^ Furthermore, d-tagatose is considered noncariogenic, has a low glycemic index of 3 (glycemic index of sucrose = 65), and is thought to be a prebiotic.^17–20^ The main industrial producers of d-tagatose are currently Bonumose, Inc. (USA), Damhert Nutrition NV (Belgium), and CJ CheilJedang (South Korea), among others.^21^ These companies use chemical and enzymatic processes with substrates such as lactose, d-galactose, d-fructose, and maltodextrin to produce d-tagatose commercially.^21^ d-galactose can be isomerized to d-tagatose by an l-AI, which is a key enzyme of the l-arabinose metabolic pathway in prokaryotes.^22^ More than 40 l-AIs have been identified and characterized from various prokaryotes in the last few decades.^23^ Overall, l-AIs showed a wide spectrum of temperature maxima (15–95 °C) and pH optima (pH 5.0–10.5).^24^ Notably, most l-AIs exhibited a maximum activity in the thermophilic temperature range of 50–70 °C.^24^ Higher temperatures (≥60 °C) favor the isomerization equilibrium toward d-tagatose formation, thereby increasing the product yield.^25^ In addition, the activity and thermostability of these enzymes were either dependent on or enhanced by divalent metal ions, particularly cobalt (Co^2+^) or manganese (Mn^2+^) ions.^24^ Furthermore, the majority of l-AIs showed a maximum activity at neutral to slightly alkaline pH (pH 7–8), while their activity and stability were low at a pH ≤ 5.^24^ As a result, the isomerization of d-galactose by an l-AI has, so far, been limited to dairy byproducts with a neutral pH.^26–28^ These studies investigated the bienzymatic conversion of lactose in cheese whey and whey permeate using a β-gal and l-AI.^26–28^

A novel l-AI from Lentilactobacillus parakefiri DSM 10551 (l-AI-Lp) was biochemically characterized and compared to a protein-engineered variant of l-AI from Geobacillus stearothermophilus DSM22 (l-AI-N13) in a previous study.^29^ The latter is part of the EP2834354B1 patent, which is owned by Damhert Nutrition.^30^ l-AI-Lp showed a maximal activity at 75 °C in the presence of CoCl_2_ and had a relative activity of ≥70% at pH 4–9.^29^ By contrast, l-AI-N13 showed no activity at pH ≤ 5.^29^ Furthermore, the two l-AIs were compared in isomerization experiments.^29^ In these experiments, 45% and 41% of 300 mM d-galactose in a buffered solution at pH 7.5 were isomerized by l-AI-Lp and l-AI-N13, respectively, after 24 h.^29^ In addition, only l-AI-Lp isomerized 45% of the galactose at pH 4.5 after 24 h.^29^

The objective of the present study was to compare l-AI-Lp and l-AI-N13 for the isomerization of d-galactose to d-tagatose in dairy byproducts, using skim milk UF-permeates at pH 6.5 and 4.5. Therefore, the influence of relevant process parameters, including metal ions and temperature, on the isomerization of d-galactose and the stability of l-AIs was systematically investigated. In addition, l-AIs were compared in bioconversion experiments using d-galactose in skim milk UF-permeate at pH 6.5 as the substrate. Finally, l-AI-Lp was applied in combination with a commercial β-gal and XI for the conversion of lactose to d-glucose, d-galactose, d-fructose, and d-tagatose in skim milk UF-permeate at pH 6.5. In addition, lactose in skim milk UF-permeate at pH 4.5 was converted to d-glucose, d-galactose, and d-tagatose by a commercial β-gal and l-AI-Lp. This study is the first to demonstrate the enzymatic conversion of lactose to d-glucose, d-galactose, and d-tagatose in dairy byproducts at both neutral and acidic pH, enabling the potential application of the byproduct as a sucrose substitute in food products. This approach demonstrates an innovative solution for valorizing dairy byproducts while supporting the development of sustainable sweetening solutions.

## 2 Materials and Methods

### 2.1 Chemicals, Materials, Enzymes, and Apparatus

All chemicals were of analytical grade and were purchased from the following companies: Carl Roth GmbH (Karlsruhe, Germany), Merck KGaA (Darmstadt, Germany), Thermo Fisher Scientific (Schwerte, Germany), and Th. Geyer GmbH (Renningen, Germany). The bovine serum albumin (modified Cohn Fraction V, pH 5.2) was obtained from SERVA Electrophoresis GmbH (Heidelberg, Germany). Precision Plus Protein unstained protein standard (10–250 kDa) was purchased from Bio-Rad Laboratories GmbH (Feldkirchen, Germany). PD-10 and MidiTrap G-25 columns were obtained from GE Healthcare (München, Germany). Amicon Ultra-15 centrifugal filters (molecular weight cutoff of 10 kDa) were obtained from Merck (Darmstadt, Germany). Solid phase extraction columns (Chromabond PS-Mix, 100 μm) were purchased from Macherey-Nagel (Düren, Germany). Skim milk UF-permeate (pH 4.5 and 6.5) was kindly provided by the Department of Soft Matter Science and Dairy Technology (University of Hohenheim, Stuttgart, Germany). The composition of the skim milk UF-permeates was determined by the Core Facility Hohenheim (Stuttgart, Germany) and is shown in Table S1. Hexokinase/Glucose-6-phosphate dehydrogenase was purchased from Megazyme International Ireland (Wicklow, Ireland). The β-gal preparation Saphera 2600 L was kindly provided by Novozymes (Bagsværd, Denmark). The β-gal preparation opti-lactase green acid line 10.000 and XI preparation opti-zym GI2 were kindly provided by Optiferm GmbH (Oy-Mittelberg, Germany). The ultrasonic processor UP200S from Hielscher Ultrasonics GmbH (Teltow, Germany) was used to disrupt the cells. High-performance liquid chromatography (HPLC) analysis was done on an Agilent 1260 infinity II LC system (Agilent Technology, Santa Barbara, California).

### 2.2 Recombinant Production and Partial Purification of l-AIs

The recombinant Escherichia coli BL21­(DE3) pET20b_l-*AI-Lp* and E. coli BL21­(DE3) pET20b_l-*AI-N13* strains were obtained from the work of Weber et al.^29^ These strains were cultivated in LB-SB medium (LB medium supplemented with d-sorbitol and betaine) using shake flasks according to the method of Weber et al., to produce l-AI-Lp and l-AI-N13.^29^ The resulting cell pellets were resuspended in either 2-(*N*-morpholino)­ethanesulfonic acid (MES) buffer (25 mM, pH 6.5 with 1 mM CoCl_2_) in the case of l-AI-Lp or tris­(hydroxymethyl)­aminomethane buffer (25 mM, pH 7.5 with 1 mM MnCl_2_) for the l-AI-N13 to a 30% (w/v) cell suspension. Both l-AIs were then partially purified by heat treatment, as described by Weber et al. and desalted using PD-10 columns to MES buffer (25 mM, pH 6.5) to remove unbound Co^2+^ or Mn^2+^ ions.^29^ The l-AI solutions were then concentrated 10–15-fold using Amicon Ultra-15 centrifugal filters with a molecular weight cutoff of 10 kDa. The protein concentration of the l-AI solutions was quantified according to Bradford, using bovine serum albumin as the standard.^31^ The purity of the partially purified l-AI-Lp and l-AI-N13 was compared with commercial β-gal and XI preparations by sodium dodecyl sulfate-polyacrylamide gel electrophoresis (SDS-PAGE) on an 8% separating gel according to Weber et al.^29^ The partially purified l-AI solutions were stored at −20 °C until further use.

### 2.3 Standard Enzyme Activity Assays

The l-AI and XI activity was measured on a 1 mL scale according to the principle of the method described by Zhang et al.^32^ d-galactose and d-glucose (300 mM) dissolved in simulated milk ultrafiltrate (SMUF) or skim milk UF-permeate were used as substrates. The SMUF was prepared according to Dumpler et al., with the following composition: 0.833 g/L KH_2_PO_4_, 0.753 g/L K_2_HPO_4_, 0.533 g/L K_3_C_6_H_5_O_7_·H_2_O, 1.2 g/L Na_3_C_6_H_5_O_7_·2H_2_O, 0.6 g/L Mg_3_(C_6_H_5_O_7_)_2_·9H_2_O, 0.057 g/L C_6_H_8_O_7_·H_2_O, 0.933 g/L KCl, 0.167 g/L NaCl, 0.2 g/L K_2_SO_4_, 0.933 g/L CaCl_2_·2H_2_O, and 0.056 g/L KOH.^33^ The pH of the SMUF was adjusted to pH 6.5 and 4.5 with lactic acid. In order to determine the l-AI and XI activity, substrate solutions containing 333.33 mM d-galactose or d-glucose, respectively, were preincubated at 60 °C. The isomerization was started by adding 50 μL of the enzyme solution to 450 μL of the substrate solution, followed by incubation at 60 °C for 5 min with stirring at 1000 rpm. The reaction was stopped by adding 150 μL of 2 M HCl, and then the mixture was incubated on ice for 5 min. Subsequently, the mixture was centrifuged (20,000*g*, 5 min, 4 °C) and the supernatant was diluted in ddH_2_O. The concentrations of d-tagatose and d-fructose were determined based on the method described by Weber et al. using calibration curves with mixtures of d-galactose (275–300 mM) and d-tagatose (0–25 mM) or d-glucose (275–300 mM) and d-fructose (0–25 mM), respectively.^29^ One katal was defined as the amount of enzyme that converts 1 mol of d-galactose to d-tagatose or d-glucose to d-fructose per second.

The activities of the β-gal preparations Saphera 2600 L and opti-lactase green acid line 10.000 were determined on a scale of 1.5 mL using skim milk UF-permeate at pH 6.5 (95.5 g/L lactose) and pH 4.5 (100.3 g/L lactose), respectively, as the substrate. The substrate solution (1498 μL) was preheated to 15 °C and hydrolysis was started by adding 2 μL of β-gal solution. The reaction mixture was then incubated at 15 °C, 1000 rpm for 5–15 min. Subsequently, 200 μL of the reaction mixture was added to 300 μL of HClO_4_ (1 M) to stop the hydrolysis. The solution was incubated on ice for 5 min and then centrifuged at 20,000*g* (5 min, 4 °C). Afterward, 100 μL of the supernatant was mixed with 72 μL of KOH (1 M) for neutralization. The solution was then incubated on ice for 5 min and centrifuged at 20,000*g* (5 min, 4 °C). The d-glucose concentration of the supernatant was measured enzymatically with the hexokinase/glucose-6-phosphate dehydrogenase assay, according to Lutz-Wahl et al.^34^ One katal was defined as the amount of enzyme that converts 1 mol of lactose to d-glucose per second.

### 2.4 Influence of Metal Ions and Temperature on the Isomerization of d-Galactose in SMUF

The influence of Mn^2+^ and Co^2+^ ions as well as temperature on the isomerization of d-galactose (300 mM) in SMUF at pH 6.5 and 4.5 was investigated using l-AI-Lp and l-AI-N13 (0.35 mg_protein_/mL_SMUF_). Both l-AIs were desalted to a MES buffer (25 mM, pH 6.5) containing either 1 mM CoCl_2_, MnCl_2_, or no additional ions using PD-10 columns prior to the experiments. The l-AI activities were determined under standard assay conditions in SMUF (pH 6.5 and 4.5) in the presence of 1 mM CoCl_2_, MnCl_2_, or without additional ions. The isomerization experiments were performed on a 1 mL scale. The influence of metal ions was investigated at 60 °C. The influence of temperature on isomerization by l-AI-Lp was investigated in SMUF at pH 6.5 (without adding metal ions) in the range of 60–70 °C and at pH 4.5 (with 1 mM CoCl_2_) in the range of 50–65 °C.

Samples (125 μL) were taken after 5, 8, and 24 h and immediately cooled on ice for 5 min. The samples were then centrifuged (20,000*g*, 5 min, 4 °C) and 50 μL of the supernatant was mixed with an equal volume of 1 M HCl. This mixture was centrifuged again (20,000*g*, 5 min, 4 °C) and the supernatant was diluted in ddH_2_O. The concentration of d-tagatose was measured as described by Weber et al.^29^

### 2.5 Investigation of Thermostability of l-AI-Lp and l-AI-N13 in Skim Milk UF-Permeate

The thermostability of l-AI-Lp and l-AI-N13 was investigated by incubating each enzyme (1 mg_protein_/mL_permeate_) in skim milk UF-permeate at pH 6.5 and 60 °C for 24 h. In the case of l-AI-N13, the permeate was supplemented with 1 mM MnCl_2_. In addition, the thermostability of l-AI-Lp (1 mg_protein_/mL_permeate_) in skim milk UF-permeate at pH 4.5, which was supplemented with 1 mM CoCl_2_, was determined at 60 °C for 24 h. Samples (500 μL) were taken at various time points during the incubation, placed on ice for 5 min, and then centrifuged (13,000*g*, 4 °C, 10 min). The supernatants were desalted to MES buffer (25 mM, pH 6.5) with either 1 mM CoCl_2_ for l-AI-Lp or 1 mM MnCl_2_ for l-AI-N13 using PD MidiTrap G-25 columns. The relative activities were then determined under standard assay conditions in MES buffer (25 mM, pH 6.5, 1 mM CoCl_2_) at 65 °C for l-AI-Lp and in MES buffer (25 mM, pH 6.5, 1 mM MnCl_2_) at 60 °C for l-AI-N13.

### 2.6 Analysis of the Storage Stability of l-AI-Lp

The storage stability of l-AI-Lp was analyzed by incubating l-AI-Lp solutions (7.83 mg_protein_/mL) in MES buffer (25 mM, pH 6.5), which was supplemented with 1 mM CoCl_2_, at 4 and −20 °C for 6 months. Samples were taken at 1, 2, 3, and 6 months to measure the residual l-AI activity under standard assay conditions in MES buffer (25 mM, pH 6.5, 1 mM CoCl_2_) at 65 °C. In addition, the influence of repeated freezing and thawing on l-AI activity was determined by freezing and thawing the l-AI solutions six times during 2 months of storage at −20 °C.

### 2.7 Bioconversion of d-Galactose in Skim Milk UF-Permeate at pH 6.5 by l-AIs

Bioconversion experiments were performed using 300 mM (54.05 g/L) d-galactose in skim milk UF-permeate at pH 6.5 as the substrate. The reactions were performed on a scale of 1.8 mL at 60 °C and 850 rpm for 24 h. The bioconversion experiments were standardized to an l-AI activity of 890 nkat_d‑gal, 60 °C_/g_d‑gal_ (8.4 mg_protein_/g_d‑gal_ (l-AI-Lp), 40.0 mg_protein_/g_d‑gal_ (l-AI-N13)) or a protein concentration of 40.0 mg_protein_/g_d‑gal_ (5035 nkat_d‑gal, 60 °C_/g_d‑gal_ (l-AI-Lp), 890 nkat_d‑gal, 60 °C_/g_d‑gal_ (l-AI-N13)). The applied activities of l-AI-Lp and l-AI-N13 were determined under standard assay conditions in skim milk UF-permeate (pH 6.5) and skim milk UF-permeate (pH 6.5) supplemented with 1 mM MnCl_2_, respectively. Samples (125 μL) were taken at regular intervals, incubated on ice (5 min), and prepared for the determination of the d-tagatose concentration as described in Section 2.4.

### 2.8 Influence of d-Tagatose on the l-AI Activity

The influence of d-tagatose on the activity of l-AI-Lp and l-AI-N13 in skim milk UF-permeate was investigated. Accordingly, the l-AI activity was measured under standard assay conditions in skim milk UF-permeate at pH 6.5 for l-AI-Lp and in skim milk UF-permeate at pH 6.5 supplemented with 1 mM MnCl_2_ for l-AI-N13. In both cases, the skim milk UF-permeates were supplemented with different concentrations of d-tagatose ranging from 0 to 6 mM.

### 2.9 Multienzymatic Conversion of Lactose in Skim Milk UF-Permeate at pH 6.5 and 4.5

The tri- and bienzymatic conversion of lactose in skim milk UF-permeates was done on a scale of 50 mL. The applied activities of the l-AI-Lp and the XI preparation opti-zym GI2 were determined under standard activity assay conditions in skim milk UF-permeate at pH 6.5 for the trienzymatic conversion and in skim milk UF-permeate at pH 4.5 with 1 mM CoCl_2_ for the bienzymatic conversion. The activities of the β-gal preparations Saphera 2600 L and opti-lactase green acid line 10.000 were determined as described in Section 2.3.

Regarding the trienzymatic conversion, lactose (95.5 g/L) in skim milk UF-permeate at pH 6.5 was hydrolyzed by adding 53 μL of Saphera 2600 L (1.00 μkat_lac, 15 °C_) to 49.95 mL of skim milk UF-permeate at 15 °C. After 24 h, the temperature was increased to 60 °C. Afterward, d-glucose and d-galactose were isomerized by adding 0.75 mL of opti-zym GI2 (0.53 μkat_d‑glu, 60 °C_) and 2 mL of l-AI-Lp (11.80 μkat_d‑gal_, _60 °C_) to 47.25 mL of hydrolyzed skim milk UF-permeate. The solution was then incubated at 60 °C for 24 h. Samples of 0.6 mL were taken after 24 h of hydrolysis and regularly during the isomerization. The enzymes were inactivated by heating the samples to 95 °C for 10 min. The samples were then cooled on ice for 10 min and centrifuged (20,000*g*, 4 °C, 10 min). The supernatant was diluted in ddH_2_O and 250 μL was loaded on a solid phase extraction column to remove ions. The flow-through was collected, and residual lactose and monosaccharides were eluted from the column by adding 1.5 mL of ddH_2_O. They were then quantified by HPLC.

The bienzymatic conversion of lactose (100.3 g/L) in skim milk UF-permeate at pH 4.5 was done according to the trienzymatic conversion with some modifications. The hydrolysis of lactose was started by adding 0.2 mL of opti-lactase green acid line 10.000 (9.57 μkat_lac, 15 °C_) to 49.8 mL of skim milk UF-permeate pH 4.5. The mixture was then incubated at 15 °C for 24 h. Subsequently, the temperature was increased to 60 °C and 4 mL of l-AI-Lp (13.45 μkat_d‑gal_, _60 °C_) and 0.5 mL of 100 mM CoCl_2_ dissolved in ddH_2_O were added to 45.5 mL of hydrolyzed skim milk UF-permeate. The sample taking and quantification of the sugars were done as described above.

### 2.10 HPLC Analysis of Lactose and Monosaccharides in Skim Milk UF-Permeate

HPLC was performed on an Agilent 1260 infinity II LC system (Agilent Technology, Santa Barbara, California), coupled with a 1260 infinity II refractive index detector (Agilent Technology, Santa Barbara, California) at 50 °C. A Rezex RCM-Monosaccharide Ca^2+^ (8%) column (300 × 7.8 mm, Phenomenex, Aschaffenburg, Germany) was used to separate the lactose, d-glucose, d-galactose, d-fructose, and d-tagatose. The injection volume was set to 10 μL. The separation was done at a column temperature of 75 °C and a constant flow rate of 0.5 mL/min for 35 min using ddH_2_O as the eluent (isocratic). The concentrations of lactose and the monosaccharides were quantified by using calibration curves in the range 0.19–2.9 g/L. Sorbitol was used as the internal standard at a final concentration of 2 g/L.

### 2.11 Statistical Analysis

All experiments, except the bioconversion of d-galactose in skim milk UF-permeate by l-AIs and the multienzymatic conversions of lactose, were done in triplicate. These exceptions were made in duplicate. All experiments were evaluated by determining the standard deviation in Excel (Microsoft, Redmond, Washington). Data are presented as mean values with standard deviations of less than 5%, unless otherwise stated.

## 3 Results and Discussion

The aim of this study was to investigate the potential of the previously characterized l-AI-Lp for the isomerization of d-galactose to d-tagatose in dairy byproducts and to compare its product yield with that of l-AI-N13. l-AI-Lp and l-AI-N13 were recombinantly produced in E. coli BL21­(DE3) and partially purified by heat treatment, according to the protocol of Weber et al.^29^ The purity of the l-AIs was analyzed and compared to commercial β-gal and XI preparations, which were subsequently used in the study, by using SDS-PAGE (Figure S1). The gel showed distinct bands at approximately 50 kDa, which corresponds to the theoretical molecular weights of the l-AI monomers (54–56 kDa). Afterward, the influence of metal ions and temperature on the isomerization of d-galactose by the l-AIs was investigated in SMUF, which provides a consistent and reproducible ion composition.^33^ The SMUF, established by Jenness and Koops, is an aqueous salt solution that simulates the mineral composition of milk UF-permeate.^35^ The thermostability of the l-AIs and the bioconversion of d-galactose were then performed in skim milk UF-permeate to evaluate the potential of the enzymes to produce d-tagatose under relevant application conditions.

### 3.1 Influence of Manganese and Cobalt Ions on the Isomerization of d-Galactose in SMUF

Divalent metal ions, particularly Co^2+^ and Mn^2+^ ions, are required for the (maximal) activity and thermostability of most of the biochemically characterized l-AIs.^23^ Weber et al. investigated the influence of metal salts on the activity of l-AI-Lp in MES buffer.^29^ The highest l-AI activity was measured in the presence of Co^2+^ ions.^29^ Furthermore, the production of d-tagatose by l-AI-N13 in the patent EP2834354B1 was done with Mn^2+^ ions.^30^ Therefore, one objective of the present study was to investigate the influence of Co^2+^ and Mn^2+^ ions on the isomerization of d-galactose to d-tagatose by l-AI-Lp and l-AI-N13 in SMUF at pH 6.5 and 4.5.

The volumetric activities of l-AI-Lp in SMUF at pH 6.5 were similar in both the absence and presence of Co^2+^ and Mn^2+^ ions with 28–29 nkat_d‑gal, 60 °C_/mL_SMUF_. By comparison, these metal ions increased the activity of l-AI-N13 from 0.2 (without additional ions) to 3.2 nkat_d‑gal, 60 °C_/mL_SMUF_ (in the presence of Mn^2+^ ions) and 4.0 (in the presence of Co^2+^ ions). The influence of Co^2+^ and Mn^2+^ ions on the isomerization of d-galactose by l-AI-Lp in SMUF at pH 6.5 was found to be negligible, with similar d-tagatose concentrations of 84.6–86.3 mM after 24 h (Table 1). However, the isomerization catalyzed by l-AI-N13 at the same pH in SMUF was dependent on the presence of Mn^2+^ or Co^2+^ ions. In the absence of metal ions, no d-tagatose was detected after 24 h, whereas with Mn^2+^ or Co^2+^ ions, d-tagatose was produced at concentrations of 58.3 and 55.6 mM, respectively. The metal ion dependency of l-AI-N13 is consistent with the results reported by Lee et al., who investigated the influence of ethylenediaminetetraacetic acid and metal salts on the activity of l-AI from Geobacillus stearothermophilus, which is the wild-type enzyme of l-AI-N13.^36^ In their study, a complete loss of l-AI activity was reported after incubation with ethylenediaminetetraacetic acid, while subsequent incubation with Mn^2+^ ions restored its maximal activity.^36^

**Table 1:** Influence of Co^2+^ and Mn^2+^ Ions (1 mM) on the Isomerization of d-Galactose (300 mM) to d-Tagatose by l-AI-Lp and l-AI-N13 (0.35 mg_protein_/mL_SMUF_) in SMUF at pH 6.5 and 4.5 at 60 °C

		d-tagatose [mM] produced by l-AI-Lp			d-tagatose [mM] produced by l-AI-N13		
		5 h	8 h	24 h	5 h	8 h	24 h
pH 6.5	none^a^	39.5	51.1	84.6	0.0	0.0	0.0
	Co^2+^	41.9	53.3	85.7	26.9	35.7	55.6
	Mn^2+^	41.6	52.3	86.3	25.8	34.2	58.3
pH 4.5	none^a^	6.1	8.7	8.5	0.0	0.0	0.0
	Co^2+^	38.5	48.5	73.4	0.0	0.0	0.0
	Mn^2+^	32.5	41.3	57.6	0.0	0.0	0.0

a The isomerization was done in SMUF without the addition of Co^2+^ and Mn^2+^ ions.

The activities of l-AI-Lp in SMUF at pH 4.5 were 4.5 (without additional ions), 8.2 (in the presence of Mn^2+^), and 20.0 nkat_d‑gal, 60 °C_/mL_SMUF_ (in the presence of Co^2+^). In the absence of these metal ions, the d-tagatose concentration increased only to 8.7 mM and stagnated after 8 h. However, the addition of Co^2+^ or Mn^2+^ ions increased the d-tagatose yield to 73.4 and 57.6 mM, respectively, after 24 h. By contrast, l-AI-N13 showed no activity in SMUF at pH 4.5, regardless of the presence of Co^2+^ or Mn^2+^ ions. Therefore, no d-tagatose was detected in the presence or absence of these ions after 24 h. These results are consistent with the previously reported pH profiles of the l-AIs, where l-AI-Lp and l-AI-N13 had relative activities of 89% and 0%, respectively, in sodium acetate buffer at pH 4.5.^29^

The metal ion dependence of l-AIs has also been described in other studies. In the study by Zheng et al., Mn^2+^ ions were added to cheese whey for the isomerization of d-galactose by a protein-engineered variant of l-AI from Bacillus coagulans, as the enzyme’s activity was dependent on Mn^2+^ or Co^2+^ ions.^28^ Similarly, Cervantes et al. added Mn^2+^ ions to whey permeate for the isomerization of d-galactose by l-AI from Bacillus stearothermophilus, as Mn^2+^ supplementation increased the yield of d-tagatose by 1.4-fold.^27^

In summary, the isomerization of d-galactose in SMUF at pH 6.5 by l-AI-Lp was independent of Co^2+^ and Mn^2+^ ion supplementation. The absence of Co^2+^ and Mn^2+^ ions at pH 6.5 is advantageous for the application of l-AI-Lp, as it would reduce the purification costs associated with d-tagatose production in comparison with l-AI-N13 and other reported l-AIs. However, the addition of Co^2+^ ions to SMUF at pH 4.5 increased the yield of d-tagatose from 8.7 to 73.4 mM. In contrast, l-AI-N13 showed no activity at this pH.

### 3.2 Influence of Temperature on the Isomerization of d-Galactose in SMUF by l-AI-Lp

In addition to the influence of metal ions, the influence of the temperature on d-tagatose production by l-AI-Lp in SMUF at pH 6.5 and 4.5 was investigated. The solubility of d-galactose increases with rising temperature, with concentrations of ≈326 and ≈611 g/L_water_ at 25 and 75 °C, respectively.^37^ Moreover, temperatures ≥60 °C shift the equilibrium of the isomerization toward d-tagatose, while simultaneously reducing microbial growth in dairy-derived matrices.^25,38^ However, temperatures above 80 °C can cause Maillard reactions, resulting in the browning of the reaction medium.^39^ Despite the highest activity of l-AI-Lp in SMUF at pH 6.5 and 70 °C, l-AI-Lp showed the highest production of d-tagatose at 65 °C, reaching 95.6 mM d-tagatose after 24 h (Figure 1A). This is probably due to the lower thermostability of l-AI at 70 °C compared to 65 °C. The temperature dependence of d-tagatose production by l-AI-N13 was not analyzed, as no activity was detected in SMUF at pH 6.5 at temperatures above 60 °C. This result contrasts with the previously reported temperature profile of l-AI-N13, which was determined in 3-(*N*-morpholino)­propane-sulfonic acid buffer (pH 7.5), where relative activities of 99% and 37% were measured at 65 and 70 °C, respectively.^29^ The reason for this is probably the different ionic composition between the 3-(*N*-morpholino)­propane-sulfonic acid buffer and SMUF.

**Figure 1:**
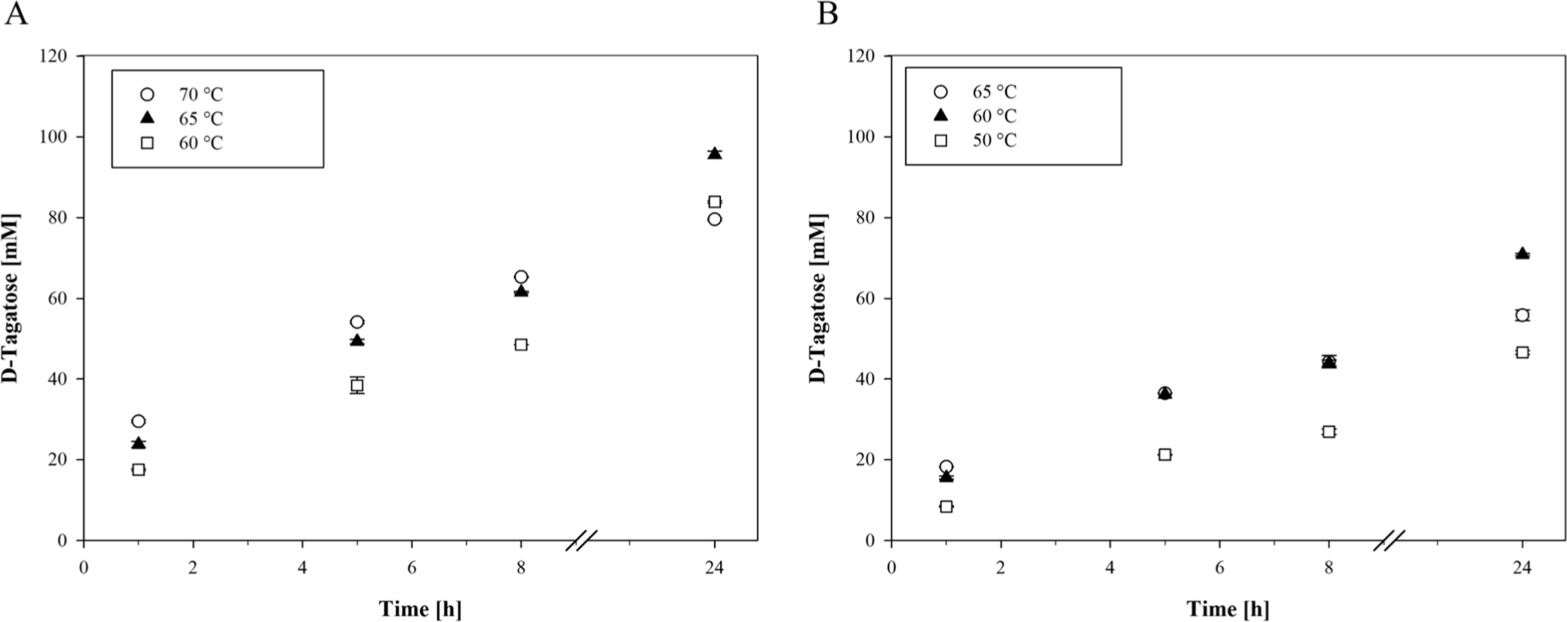
Influence of temperature on the isomerization of d-galactose to d-tagatose in SMUF by l-AI-Lp at pH 6.5 (A) and 4.5 (B). The isomerization of d-galactose (300 mM) was done using 0.35 mg_protein_/mL_SMUF_ l-AI-Lp. SMUF at pH 4.5 was supplemented with 1 mM CoCl_2_. The applied l-AI activities in SMUF at pH 6.5 were 29.0 (60 °C), 39.8 (65 °C), and 48.1 nkat_d‑gal_/mL_SMUF_ (70 °C). The applied activities of l-AI-Lp in SMUF at pH 4.5 were 5.1 (50 °C), 18.6 (60 °C), and 20.5 nkat_d‑gal_/mL_SMUF_ (65 °C).

Furthermore, the influence of temperature on the production of d-tagatose by l-AI-Lp in SMUF at pH 4.5 with Co^2+^ ions was analyzed over a temperature range of 50–65 °C. Although the volumetric activity of l-AI-Lp at this pH increased with temperature, the concentration of d-tagatose after 8 h was similar (approximately 44 mM) at 60 and 65 °C (Figure 1B). Afterward, the d-tagatose concentration increased to 70.9 mM at 60 °C and to 55.8 mM at 65 °C after 24 h, probably due to the higher thermostability of l-AI-Lp at 60 °C compared to 65 °C. Consequently, the influence of temperatures above 65 °C was not further investigated. No activity was detected for l-AI-N13 in SMUF at pH 4.5 supplemented with 1 mM MnCl_2_, irrespective of the temperature.

The influence of temperature on the isomerization of d-galactose by l-AI from Enterococcus faecium was investigated in the study by Torres and Batista-Viera.^13^ Therefore, l-AI was coimmobilized with the β-gal from Bacillus circulans to produce d-tagatose from lactose in demineralized cheese whey.^13^ The isomerization increased with temperature, from 29% at 30 °C to 45% at 50 °C.^13^ However, raising the temperature further to 60 °C did not increase the isomerization, due to the low thermostability of l-AI at this temperature.^13^ Although l-AI-Lp showed the highest isomerization at 65 °C in SMUF at pH 6.5, subsequent isomerization experiments were performed at 60 °C for a direct comparison between l-AI-Lp and l-AI-N13.

### 3.3 Investigation of the Thermostability of l-AI-Lp and l-AI-N13 in Skim Milk UF-Permeate

The thermostability of l-AI-Lp and l-AI-N13 was investigated in skim milk UF-permeate to compare their potential to isomerize d-galactose in dairy byproducts. The pH of skim milk UF-permeate was adjusted to pH 6.5 and 4.5 to simulate the pH of sweet and acid whey. Based on the previous results, the thermostability of l-AI-Lp was investigated in skim milk UF-permeate at pH 6.5 and 4.5, with the latter being supplemented with 1 mM CoCl_2_. The thermostability of l-AI-N13 was determined only in skim milk UF-permeate at pH 6.5, which was supplemented with 1 mM MnCl_2_. l-AI-Lp retained a relative activity of 75%, while l-AI-N13 had a lower relative activity of 32% at pH 6.5 after 24 h (Figure S2). Furthermore, l-AI-Lp showed relative activities of 39% and 6% after 8 and 24 h at pH 4.5, respectively (Figure S3).

The thermostability of various l-AIs has been investigated in previous studies.^36,40–42^ In the latter, the thermostabilities of l-AIs from Lactobacillus plantarum, Lactobacillus fermentum, Enterococcus faecium, and Geobacillus stearothermophilus were found to be dependent on the presence of Mn^2+^ ions.^36,40–42^ The l-AI from Lactobacillus plantarum, for instance, retained its activity after 2 h at 70 °C in the presence of Mn^2+^ ions at pH 7.5.^40^ On the contrary, in the absence of Mn^2+^ ions, l-AI was completely inactivated after 30 min^40^ In conclusion, the relatively high thermostability of l-AI-Lp in the absence of Mn^2+^ and Co^2+^ ions is advantageous for d-tagatose production at a neutral pH, compared to that of l-AI-N13 and previously characterized l-AIs.

Although the thermostability of l-AI-Lp was 2.3-fold higher than that of l-AI-N13, its stability could be further increased by the immobilization of the enzyme. Previous studies have demonstrated the immobilization of other l-AIs on alginate or acrylic beads.^43–45^ The immobilization of the l-AI from Enterococcus faecium onto acrylic epoxy-activated beads, for instance, increased its stability from a half-life of 6 h (nonimmobilized l-AI) to 379 h (immobilized l-AI) at 50 °C.^45^

### 3.4 Analysis of the Storage Stability of l-AI-Lp

The storage stability of l-AI-Lp in MES buffer (pH 6.5) containing 1 mM CoCl_2_ was investigated at 4 and −20 °C. The l-AI activity decreased to 75% ± 4% at 4 °C and 97% ± 4% at −20 °C after 6 months (Table 2). Furthermore, the influence of freezing and thawing on the activity of l-AI-Lp was investigated. The l-AI-Lp solutions were frozen and thawed six times over 2 months, and a residual activity of 103% ± 2% was measured after the last thawing (data not shown). l-AI-Lp showed a relatively high storage stability at 4 °C compared to other l-AIs characterized. The stability of l-AI from Lactobacillus fermentum in phosphate buffer (pH 6.0) at 4 °C was investigated in another study and l-AI retained 88% of its initial activity after 24 h.^41^ Furthermore, the stabilities of the l-AIs from Lactobacillus sakei and Shewanella sp. ANA-3 in MES buffer (pH 6.5) at 4 °C were analyzed and a relative activity of 50% was determined for both after 43 h.^46,47^ The storage stability of l-AI-Lp could be further increased by adding cryoprotectants (e.g., glycerol) to the l-AI sample.^48^

**Table 2:** Storage Stability of l-AI-Lp in the MES Buffer (25 mM, pH 6.5 + 1 mM CoCl_2_)

temperature [°C]	storage time [months]	residual activity [%]^a^
4	1	101 ± 4
	2	92 ± 1
	3	83 ± 2
	6	75 ± 4
–20	1	100 ± 2
	2	100 ± 1
	3	96 ± 1
	6	97 ± 4

a 100% = 1140.9 ± 23.2 nkat_d‑gal, 65 °C_/mL.

### 3.5 Bioconversion of d-Galactose in Skim Milk UF-Permeate by l-AI-Lp and l-AI-N13

l-AI-Lp and l-AI-N13 were compared in bioconversion experiments to evaluate their potential for the isomerization of d-galactose to d-tagatose in skim milk UF-permeate at a neutral pH. The experiments were done with 300 mM (54.05 g/L) d-galactose in skim milk UF-permeate at pH 6.5 at 60 °C, using either an equal l-AI activity (890 nkat_d‑gal, 60 °C_/g_d‑gal_) (Figure 2A) or an equal protein concentration (40 mg_protein_/g_d‑gal_) (Figure 2B). The concentration of d-tagatose in the activity-standardized bioconversion increased to 99.3 ± 0.1 mM with l-AI-Lp and 135.7 ± 0.1 mM with l-AI-N13 after 24 h (Figure 2A), corresponding to d-galactose isomerizations of 33% and 45%, respectively. Although l-AI-Lp showed a 2.3-fold higher thermostability than l-AI-N13 under bioconversion conditions, the yield of d-tagatose was 1.3-fold lower compared to l-AI-N13. The reason for this is probably the decrease in the l-AI-Lp activity in the presence of relatively low concentrations of d-tagatose (≥2 mM). This hypothesis was validated by additional experiments comparing the influence of d-tagatose concentrations (0–6 mM) on the activity of l-AI-Lp and l-AI-N13 (Figure S4). The relative activity of l-AI-Lp decreased to 53% in the presence of 2 mM d-tagatose, whereas l-AI-N13 retained full activity under the same conditions.

**Figure 2:**
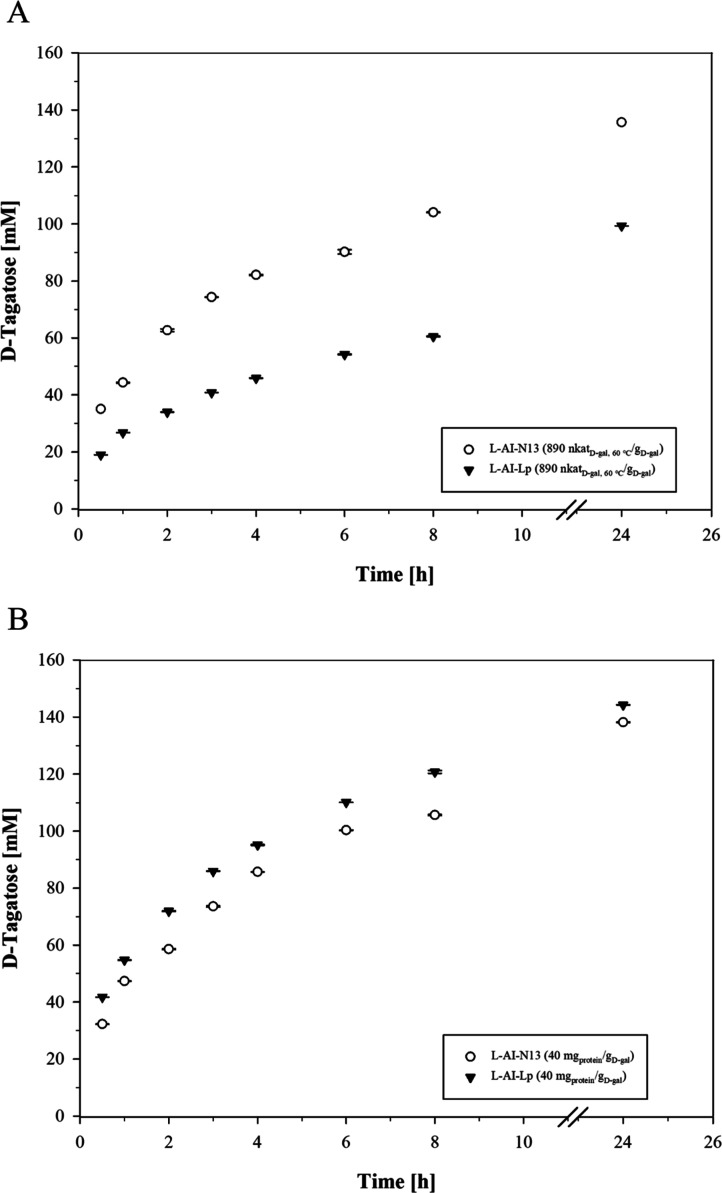
Bioconversion of d-galactose (300 mM) to d-tagatose in skim milk UF-permeate (pH 6.5) by l-AI-Lp and l-AI-N13 at 60 °C (working volume 1.8 mL). The bioconversion experiments were done with a standardized l-AI activity of 890 nkat_d‑gal, 60 °C_/g_d‑gal_ (A) or protein concentration of 40 mg_protein_/g_d‑gal_ (B). In the case of l-AI-N13, 1 mM MnCl_2_ was added to skim milk UF-permeate.

Although the bioconversion experiments were standardized to equal l-AI activity, the protein concentration of l-AI-Lp was 4.8-fold lower than that of l-AI-N13. Therefore, an additional bioconversion was done using equal protein concentrations with l-AI activities of 5035 (l-AI-Lp) and 890 nkat_d‑gal, 60 °C_/g_d‑gal_ (l-AI-N13). The concentration of d-tagatose increased within 24 h to 144.3 ± 0.1 mM with l-AI-Lp and 138.2 ± 0.1 mM with l-AI-N13, corresponding to d-galactose isomerizations of 48% and 46%, respectively (Figure 2B). After 24 h, a further l-AI activity of 109 nkat_d‑gal, 60 °C_ was added to the bioconversion with l-AI-Lp to investigate whether the chemical equilibrium had been reached. After 6 h, the isomerization stagnated at 49.6% ± 0.3% (data not shown), indicating that the equilibrium of the isomerization of d-galactose to d-tagatose under these bioconversion conditions is approximately 50%. The equilibrium could be shifted to higher d-tagatose concentrations by adding boric acid to the reaction mixture, which forms a complex with d-tagatose.^28^ In previous studies, the isomerization yield of d-tagatose at 50–60 °C increased from approximately 50% in the absence of boric acid to 77%–83% in its presence.^28,49^

In summary, the same protein concentration of l-AI-Lp and l-AI-N13 yielded a similar concentration of d-tagatose in skim milk UF-permeate at pH 6.5. The d-galactose isomerization by these l-AIs is higher than the results reported in other studies, where an l-AI isomerized 22%–45% of d-galactose in whey or whey permeate.^13,26–28^ Furthermore, it is noteworthy that the isomerization by l-AI-Lp was done without the addition of metal ions, particularly Mn^2+^ ions, in contrast to the isomerization by l-AI-N13 and other l-AIs used in the studies by Cervantes et al. and Zheng et al.^27,28^ In the former, d-galactose (500 mM) in whey permeate (pH 7.5, 0.5 mM Mn^2+^ ions) was isomerized by l-AI from Bacillus stearothermophilus (120 nkat_d‑gal, 65 °C_/g_d‑gal_) at 65 °C.^27^ The isomerization of d-galactose increased to a maximum of 33% after 7 h.^27^ In the study by Zheng et al., d-galactose (291 mM) in cheese whey (pH 6.0, 1 mM Mn^2+^ ions) was isomerized to 130 mM d-tagatose by a protein-engineered variant of l-AI from Bacillus coagulans (440 nkat_d‑gal, 50 °C_/g_d‑gal_) at 50 °C after 12 h.^27^ This reaction resulted in a maximum d-galactose isomerization of approximately 45%.^28^

### 3.6 Multienzymatic Conversion of Lactose in Skim Milk UF-Permeate at pH 6.5 and 4.5

Finally, the multienzymatic conversions of lactose in skim milk UF-permeate at pH 6.5 and 4.5 were investigated using commercial β-gal, XI preparations, and l-AI-Lp on a 50 mL scale. In the first step, lactose was hydrolyzed to d-glucose and d-galactose using the β-gal preparations Saphera 2600 L at pH 6.5 and opti-lactase green acid-line 10.000 at pH 4.5. Subsequently, d-galactose was isomerized to d-tagatose by l-AI-Lp and d-glucose was simultaneously isomerized to d-fructose using the XI preparation opti-zym GI2. However, the application of opti-zym GI2 was limited to pH 6.5, since no XI activity was detected in skim milk UF-permeate at pH 4.5.

In skim milk UF-permeate at pH 6.5 (95.5 g/L lactose), 97.6% ± 0.1% lactose was hydrolyzed by a Saphera 2600 L at 15 °C after 24 h. The products d-glucose and d-galactose were then simultaneously isomerized to d-fructose and d-tagatose by opti-zym GI2 and l-AI-Lp at 60 °C, respectively (Figure 3). Amounts of 23.7 ± 0.1 g/L of d-fructose and 23.2 ± 0.1 g/L of d-tagatose were produced after 24 h, corresponding to d-glucose and d-galactose isomerizations of 51% and 50%, respectively. In addition, the lactose hydrolysis increased to 98.2% ± 0.1% simultaneously with the isomerization. In the end, 22.9 ± 0.1 g/L of d-glucose, 22.8 ± 0.1 g/L of d-galactose, 23.7 ± 0.1 g/L of d-fructose, and 23.2 ± 0.1 g/L of d-tagatose were produced.

**Figure 3:**
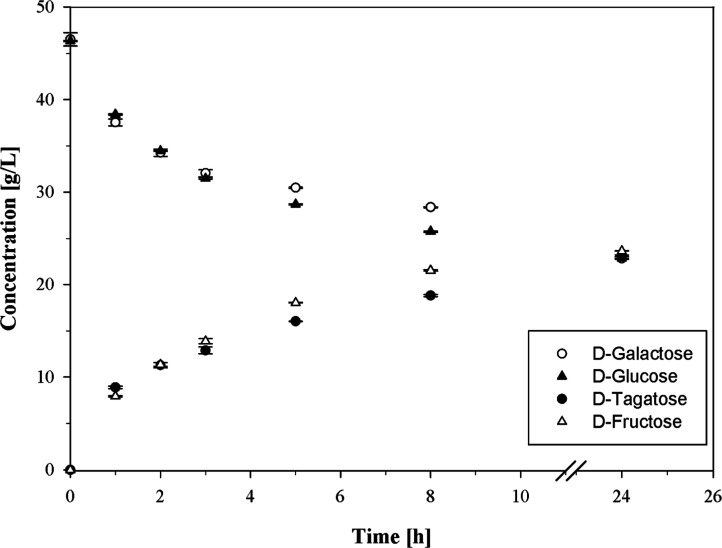
Simultaneous isomerization of d-galactose to d-tagatose and d-glucose to d-fructose in skim milk UF-permeate at pH 6.5 (working volume 50 mL). d-galactose (46.5 g/L) and d-glucose (46.4 g/L) were isomerized by l-AI-Lp (230 nkat_d‑gal, 60 °C_/mL_permeate_) and opti-zym GI2 (11 nkat_d‑glu, 60 °C_/mL_permeate_) at 60 °C, respectively.

In an additional experiment, 96.1% ± 0.1% of the lactose in skim milk UF-permeate at pH 4.5 (100.3 g/L lactose) was hydrolyzed by opti-lactase green acid line 10.000 at 15 °C after 24 h. Subsequently, d-galactose was isomerized to d-tagatose by l-AI-Lp at 60 °C (Figure 4). An amount of 22.7 ± 0.2 g/L d-tagatose was produced after 24 h, corresponding to a d-galactose isomerization of 50%. Simultaneously with isomerization, the lactose hydrolysis increased to 98.6% ± 0.1%. d-glucose was not isomerized as no XI activity was detected for opti-zym GI2 in skim milk UF-permeate at pH 4.5. In the end, 46.1 ± 0.5 g/L of d-glucose, 22.4 ± 0.1 g/L of d-galactose, and 22.7 ± 0.2 g/L of d-tagatose were produced.

**Figure 4:**
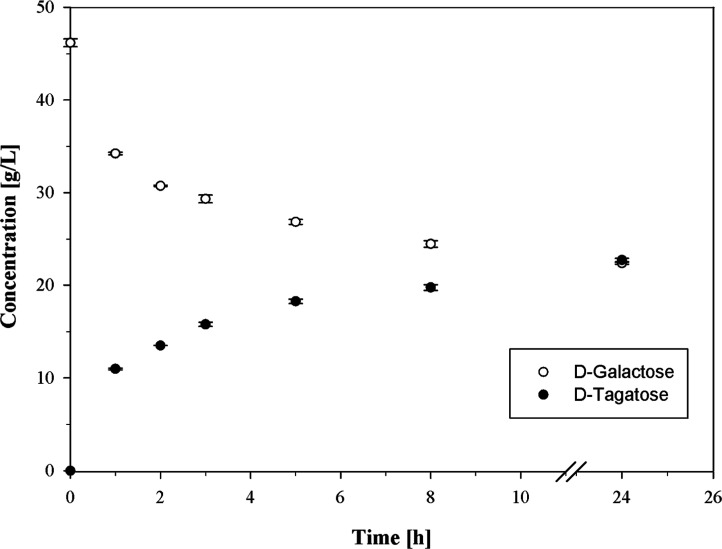
Isomerization of d-galactose to d-tagatose in skim milk UF-permeate at pH 4.5 with 1 mM CoCl_2_ (working volume 50 mL). d-galactose (46.2 g/L) was isomerized by l-AI-Lp (269 nkat_d‑gal, 60 °C_/mL_permeate_) at 60 °C.

The isomerization of d-galactose to d-tagatose in a dairy byproduct has been shown in previous studies only at a neutral pH.^12,13,26–28^ The β-gal from Bacillus circulans, the XI from Streptomyces rubiginosus, and the l-AI from Enterococcus faecium were used in the study from Torres and Batista-Viera as nonimmobilized and coimmobilized enzymes for the conversion of 46 g/L lactose in 300 mL of mozzarella cheese whey (pH 7.0) at 50 °C.^13^ When the nonimmobilized enzymes were applied, a maximum of 76% of the lactose was hydrolyzed, while 21% of d-glucose and 22% of d-galactose were isomerized after 6 h.^13^ However, coimmobilization of the enzymes onto epoxy-activated acrylic beads resulted in complete lactose hydrolysis (100%) and increased isomerization yields of 39% for d-glucose and 45% for d-galactose.^13^ These isomerization yields are lower than those observed in the present study. Nevertheless, a direct comparison is not possible due to missing information about enzyme activities used in the previous study. Whole cells of Corynebacterium glutamicum were engineered in another study to convert lactose in concentrated cheese whey permeate (pH 7.5) to a mixture of d-glucose, d-galactose, d-fructose, and d-tagatose.^12^ These cells expressed the β-gal from Streptococcus thermophilus to hydrolyze lactose, the XI from Arthrobacter sp. NRRL to isomerize d-glucose and the l-AI from Bacillus coagulans to isomerize d-galactose.^12^ An amount of 98 g/L of lactose in whey permeate was converted by the engineered Corynebacterium glutamicum cells to a maximum of 24 g/L of d-glucose, 26 g/L of d-galactose, 24 g/L of d-fructose, and 20 g/L of d-tagatose after 48 h at 60 °C.^12^ This corresponds to isomerization yields of 49% for d-glucose and 44% for d-galactose.^12^ The latter yield is lower compared to the results of the present study.

In summary, l-AI-Lp was applied combined with a commercial β-gal and XI to convert 96 g/L lactose in skim milk UF-permeate at pH 6.5 to d-glucose, d-galactose, d-fructose, and d-tagatose (23–24 g/L each). Thus, l-AI-Lp showed a relatively high isomerization of d-galactose (50%) in a dairy byproduct at a neutral pH without additional metal ions. By contrast, the isomerization by l-AI-N13, similar to other reported l-AIs, required the addition of Mn^2+^ ions and was limited to neutral pH conditions. Furthermore, this study is the first to demonstrate the enzymatic conversion of lactose to d-glucose, d-galactose, and d-tagatose in a dairy byproduct at an acidic pH, yielding a relatively high isomerization of 50% using a commercial β-gal and l-AI-Lp. In conclusion, l-AI-Lp showed advantageous characteristics for producing d-tagatose in dairy byproducts compared to l-AI-N13 at both acidic and neutral pH. Furthermore, the multienzymatic conversions of lactose are promising methods to increase the sweetness of dairy byproducts with neutral or acidic pH, thereby expanding their potential use as alternative sweeteners to sucrose in the food industry. These sweetness-enhanced byproducts could be processed further into sweet syrups with potential applications, particularly in the baking industry. However, for broader industrial applicability, the cobalt dependency of l-AI-Lp at an acidic pH should be changed to a more favorable dependence on magnesium or calcium by protein engineering to reduce health concerns associated with cobalt exposure. In addition, future research should focus on the recombinant production of l-AI-Lp in microbial hosts that have a qualified presumption of safety, as defined by the European Food Safety Authority.

## Supplementary Material



## Data Availability

The data underlying this study are available in the published article and its Supporting Information.

## Notes

Composition of skim milk UF-permeate at pH 6.5 and 4.5; SDS-PAGE analysis of L-AI-Lp, L-AI-N13, and commercial enzyme preparations; thermostability of L-AI-Lp and L-AI-N13AI in skim milk UF-permeate; and influence of d-tagatose concentrations on the activity of L-AI-Lp and L-AI-N13 (PDF)

## Abbreviations

d-gal: d-galactose
d-glu: d-glucose
E. coli: Escherichia coli
HPLC: high-performance liquid chromatography
Lac: lactose
l-AI: l-arabinose isomerase
L-AI-Lp: l-arabinose isomerase from Lentilactobacillus parakefiri DSM 10551
L-AI-N13: protein-engineered variant of l-arabinose isomerase from Geobacillus stearothermophilus DSM22
MES: 2-(*N*-morpholino)­ethanesulfonic acid
RS: relative sweetness
UF-permeate: ultrafiltration permeate
SDS-PAGE: sodium dodecyl sulfate-polyacrylamide gel electrophoresis
SMUF: simulated milk ultrafiltrate
XI: xylose isomerase
β-gal: β-galactosidase

## References

[1] Eurostat . Milk and milk product statistics. https://ec.europa.eu/eurostat/statistics-explained/index.php?title=Milk_and_milk_product_statistics#SE_MAIN_TT%20/?zx=1748361754421&no_sw_cr=1 (accessed June 3, 2025).

[2] Federal Office for Agriculture and Food . Production of selected dairy products by month. https://www.ble.de/DE/BZL/Daten-Berichte/Milch-Milcherzeugnisse/_functions/TabelleMonatlicheErgebnisse2024.html?nn=623806 (accessed June 3, 2025).

[3] Peri C., Pompei C., Rossi F. (1973). Process optimization
in skim milk
protein recovery and purification by ultrafiltration. J. Food Sci..

[4] Prazeres A. R., Carvalho F., Rivas J. (2012). Cheese whey
management: A review. J. Environ. Manage..

[5] Fischer C., Kleinschmidt T. (2021). Valorisation
of sweet whey by fermentation with mixed
yoghurt starter cultures with focus on galactooligosaccharide synthesis. Int. Dairy J..

[6] Frenzel M., Zerge K., Clawin-Rädecker I., Lorenzen P. C. (2015). Comparison
of the galacto-oligosaccharide forming activity of different β-galactosidases. LWT--Food Science and Technology.

[7] Kilara, A. ; Vaghela, M. N. Whey proteins. Proteins in Food Processing, 2nd ed.; Yada, R. Y. , Ed.; Woodhead Publishing: Sawston, UK, 2018; pp 93–126.

[8] Pandey A., Srivastava S., Rai P., Duke M. (2019). Cheese whey to biohydrogen
and useful organic acids: A non-pathogenic microbial treatment by
L. acidophilus. Sci. Rep..

[9] Rocha-Mendoza D., Kosmerl E., Krentz A., Zhang L., Badiger S., Miyagusuku-Cruzado G., Mayta-Apaza A., Giusti M., Jiménez-Flores R., García-Cano I. (2021). Invited review:
Acid whey trends and health benefits. J. Dairy
Sci..

[10] Luzzi G., Steffens M., Clawin-Rädecker I., Hoffmann W., Franz C. M. A. P., Fritsche J., Lorenzen P. C. (2020). Enhancing the sweetening
power of lactose by enzymatic modification in the reformulation of
dairy products. Int. J. Dairy Technol..

[11] Lorenzen P. C., Breiter J., Clawin-Rädecker I., Dau A. (2013). A novel bi-enzymatic
system for lactose conversion. Int. J. Food
Sci. Technol..

[12] Shen J., Chen J., Jensen P. R., Solem C. (2019). Sweet as sugar –
Efficient conversion of lactose into sweet sugars using a novel whole-cell
catalyst. J. Agric. Food Chem..

[13] Torres P., Batista-Viera F. (2019). Production
of D-tagatose and D-fructose from whey by
co-immobilized enzymatic system. Mol. Catal..

[14] Hirst E. L., Hough L., Jones J. K. N. (1949). Composition
of the gum of Sterculia
setigera: Occurrence of D-tagatose in nature. Nature.

[15] Fujimaru T., Park J. H., Lim J. (2012). Sensory characteristics and relative
sweetness of tagatose and other sweeteners. J. Food Sci..

[16] Turck D., Bresson J., Burlingame B., Fairweather-Tait S., Heinonen M., Hirsch-Ernst K. I., Mangelsdorf I., McArdle H. J., Naska A., Nowicka G., Pentieva K., Sanz Y., Siani A., Sjödin A., Stern M., Tomé D., Van Loveren H., Vinceti M., Willatts P., Neuhäuser-Berthold M. (2016). Scientific
opinion on the energy conversion factor of D-tagatose for labelling
purposes. EFSA J..

[17] EFSA
Panel on Dietetic Products Nutrition and Allergies NDA (2011). Scientific Opinion on the substantiation
of health claims related to the sugar replacers xylitol, sorbitol,
mannitol, maltitol, lactitol, isomalt, erythritol, D-tagatose, isomaltulose,
sucralose and polydextrose and maintenance of tooth mineralisation
by decreasing tooth demineralisation (ID 463, 464, 563, 618, 647,
1182, 1591, 2907, 2921, 4300), and reduction of post-prandial glycaemic
responses (ID 617, 619, 669, 1590, 1762, 2903, 2908, 2920) pursuant
to Article 13(1) of Regulation (EC) No 1924/2006. EFSA J..

[18] Ahmed A., Khan T. A., Dan Ramdath D., Kendall C. W. C., Sievenpiper J. L. (2022). Rare sugars
and their health effects in humans: A systematic review and narrative
synthesis of the evidence from human trials. Nutr. Rev..

[19] Atkinson F. S., Foster-Powell K., Brand-Miller J. C. (2008). International tables of glycemic
index and glycemic load values: 2008. Diabetes
Care.

[20] Venema K., Vermunt S. H. F., Brink E. J. (2005). D-tagatose
increases butyrate production
by the colonic microbiota in healthy men and women. Microb. Ecol. Health Dis..

[21] Zhang H., Mao X., Lu Z., Gao C., Chen Z., Liu J. (2025). Advances in
biological production of D-tagatose: A comprehensive overview. Fermentation.

[22] Lepesant J. A., Dedonder R. (1967). Metabolism of L-arabinose
in Bacillus subtilis Marburg
ind-168. C. R. Hebd. Seances Acad. Sci..

[23] Miao P., Wang Q., Ren K., Zhang Z., Xu T., Xu M., Zhang X., Rao Z. (2023). Advances and prospects of D-tagatose
production based on a biocatalytic isomerization pathway. Catalysts.

[24] Roy S., Chikkerur J., Roy S. C., Dhali A., Kolte A. P., Sridhar M., Samanta A. K. (2018). Tagatose as a potential nutraceutical:
Production, properties, biological roles, and applications. J. Food Sci..

[25] Hong Y. H., Lee D. W., Lee S. J., Choe E. A., Kim S. B., Lee Y. H., Cheigh C. I., Pyun Y. R. (2007). Production of D-tagatose
at high temperatures using immobilized Escherichia coli cells expressing
L-arabinose isomerase from Thermotoga neapolitana. Biotechnol. Lett..

[26] Wanarska M., Kur J. (2012). A Method for the production of D-tagatose
using a recombinant Pichia
pastoris strain secreting β-D-galactosidase from Arthrobacter
chlorophenolicus and a recombinant L-arabinose isomerase from Arthrobacter
sp. 22c. Microb. Cell Fact..

[27] Cervantes F. V., Neifar S., Merdzo Z., Viña-Gonzalez J., Fernandez-Arrojo L., Ballesteros A. O., Fernandez-Lobato M., Bejar S., Plou F. J. (2020). A three-step process for the bioconversion
of whey permeate into a glucose-free D-tagatose syrup. Catalysts.

[28] Zheng Z., Xie J., Liu P., Li X., Ouyang J. (2019). Elegant and efficient
biotransformation for dual production of D-tagatose and bioethanol
from cheese whey powder. J. Agric. Food Chem..

[29] Weber N., Lutz-Wahl S., Fischer L. (2025). Recombinant production and characterization
of a novel L-arabinose isomerase for the production of D-tagatose
at acidic pH. Food Biosci..

[30] François, J. M. ; Counson, M. J.-P. M. ; Bouleau, C. A. N. T. ; Brans, A. F. F. ; Demarcelle, M. A. J. ; Hendrickx, W. G. J. ; Mirzaian, H. ; Fortunato, A. ; Freichels, R. ; Vastenavond, C. M. Improved galactose isomerases and use thereof in the production of tagatose. EP 2834354 B1, 2013.

[31] Bradford M. M. (1976). A rapid
and sensitive method for the quantitation of microgram quantities
of protein utilizing the principle of protein-dye binding. Anal. Biochem..

[32] Zhang Z., Wang H., Yang R., Jiang X. (2010). A novel spectrophotometric
method for quantitative determination of lactulose in food industries. Int. J. Food Sci. Technol..

[33] Dumpler J., Kieferle I., Wohlschläger H., Kulozik U. (2017). Milk ultrafiltrate
analysis by ion chromatography and calcium activity for SMUF preparation
for different scientific purposes and prediction of its supersaturation. Int. Dairy J..

[34] Lutz-Wahl S., Mozer H., Kussler A., Schulz A., Seitl I., Fischer L. (2024). A new β-galactosidase
from Paenibacillus wynnii
with potential for industrial applications. J. Dairy Sci..

[35] Jenness R., Koops J. (1962). Preparation and properties of a salt solution which simulates milk
ultrafiltrate. Neth. Milk Dairy J..

[36] Lee D. W., Choe E. A., Kim S. B., Eom S. H., Hong Y. H., Lee S. J., Lee H. S., Lee D. Y., Pyun Y. R. (2005). Distinct
metal dependence for catalytic and structural functions in the L-arabinose
isomerases from the mesophilic Bacillus halodurans and the thermophilic
Geobacillus stearothermophilus. Arch. Biochem.
Biophys..

[37] Jónsdóttir S. O. ´., Cooke S. A., Macedo E. A. (2002). Modeling and measurements of solid-liquid
and vapor-liquid equilibria of polyols and carbohydrates in aqueous
solution. Carbohydr. Res..

[38] Kamala, K. ; Kumar, V. P. Food products and food contamination. In Microbial Contamination and Food Degradation; Holban, A. M. , Grumezescu, A. M. , Eds.; Academic Press: Camridge, MA, 2018; pp 1–19.

[39] Kwon S. Y., Baek H. H. (2014). Effects of temperature, pH, organic acids, and sulfites
on tagatose browning in solutions during processing and storage. Food Sci. Biotechnol..

[40] Chouayekh H., Bejar W., Rhimi M., Jelleli K., Mseddi M., Bejar S. (2007). Characterization of an L-arabinose
isomerase from the Lactobacillus
plantarum NC8 strain showing pronounced stability at acidic pH. FEMS Microbiol. Lett..

[41] Xu Z., Qing Y., Li S., Feng X., Xu H., Ouyang P. (2011). A novel L-arabinose
isomerase from Lactobacillus fermentum
CGMCC2921 for D-tagatose production: Gene cloning, purification and
characterization. J. Mol. Catal. B:Enzym..

[42] Manzo R. M., Antunes A. S. L. M., de Sousa Mendes J., Hissa D. C., Gonçalves L. R. B., Mammarella E. J. (2019). Biochemical characterization of heat-tolerant
recombinant L-arabinose isomerase from Enterococcus faecium DBFIQ
E36 strain with feasible applications in D-tagatose production. Mol. Biotechnol..

[43] Kim H.-J., Ryu S.-A., Kim P., Oh D.-K. (2003). A feasible
enzymatic
process for D-tagatose production by an immobilized thermostable L-arabinose
isomerase in a packed-bed bioreactor. Biotechnol.
Prog..

[44] Lim B. C., Kim H. J., Oh D. K. (2008). Tagatose
production with pH control
in a stirred tank reactor containing immobilized L-arabinose isomerase
from Thermotoga neapolitana. Appl. Biochem.
Biotechnol..

[45] Torres P., Batista-Viera F. (2017). Immobilized
trienzymatic system with enhanced stabilization
for the biotransformation of lactose. Molecules.

[46] Rhimi M., Ilhammami R., Bajic G., Boudebbouze S., Maguin E., Haser R., Aghajari N. (2010). The acid tolerant L-arabinose
isomerase from the food grade Lactobacillus sakei 23K Is an attractive
D-tagatose producer. Bioresour. Technol..

[47] Rhimi M., Bajic G., Ilhammami R., Boudebbouze S., Maguin E., Haser R., Aghajari N. (2011). The acid-tolerant
L-arabinose
isomerase from the mesophilic Shewanella sp. ANA-3 is highly active
at low temperatures. Microb. Cell Fact..

[48] Vagenende V., Yap M. G. S., Trout B. L. (2009). Mechanisms of protein
stabilization
and prevention of protein aggregation by glycerol. Biochemistry.

[49] Lim B. C., Kim H. J., Oh D. K. (2007). High production of D-tagatose by
the addition of boric acid. Biotechnol. Prog..

