# Supplementary material for: Comparison of Two l‑Arabinose Isomerases for Multienzymatic Conversion of Lactose in Skim Milk Permeate at Neutral and Acidic pH
Source: J Agric Food Chem. 2025 Jun 12;73(25):15889–99. doi: 10.1021/acs.jafc.5c04545 (PMC12203602; doi:10.1021/acs.jafc.5c04545)
Supplement: Supplementary file 1 [file jf5c04545_si_001.pdf]

Supporting Information for:

Comparison of two L-arabinose isomerases for multi-enzymatic conversion of lactose in skim milk permeate at neutral and acidic pH

Nathanael Weber [a], Sabine Lutz-Wahl [a] and Lutz Fischer [a]*

[a]University of Hohenheim, Institute of Food Science and Biotechnology,

Department of Biotechnology and Enzyme Science, Garbenstr. 25, 70599 Stuttgart, Germany

*Corresponding author

Table S1. Composition of skim milk UF-permeate pH 6.5 and 4.5.

	pH 6.5	pH 4.5
**lactose [g/L]**[a]	95.47 ± 1.57	100.30 ± 0.06
**calcium [mg/L]**[b]	241.98	354.07
**potassium [mg/L]**[b]	1940.57	2242.4
**magnesium [mg/L]**[b]	100.12	116.98
**sodium [mg/L]**[b]	536.99	531.77
**phosphorus [mg/L]**[b]	464.15	586.44
**chloride [mg/L]**[c]	1621.6	1662.0
**phosphate [mg/L]**[c]	1521.0	1600.6
**lactate [mg/L]**[c]	N.d.	1759.5

[a] Determination by the HPLC method of the present study
[b] Determination by inductively coupled plasma optical emission spectrometry
[c] Determination by ion chromatography
N.d., not determined

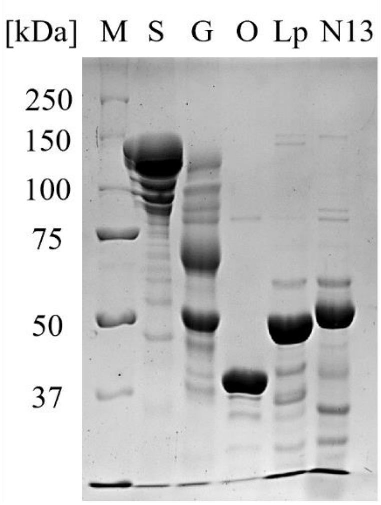

Figure S1. SDS-PAGE analysis of L-AI-Lp, L-AI-N13 and commercial enzyme preparations. M: Precision Plus Protein™ unstained protein standard; S: Saphera 2600 L; G: opti-lactase green acid-line 10.000; O: opti-zym GI2; Lp: heat treated cell-free extract containing L-AI-Lp; N13: heat treated cell-free extract containing L-AI-N13. 5 µg total protein loaded per lane, Coomassie stained.

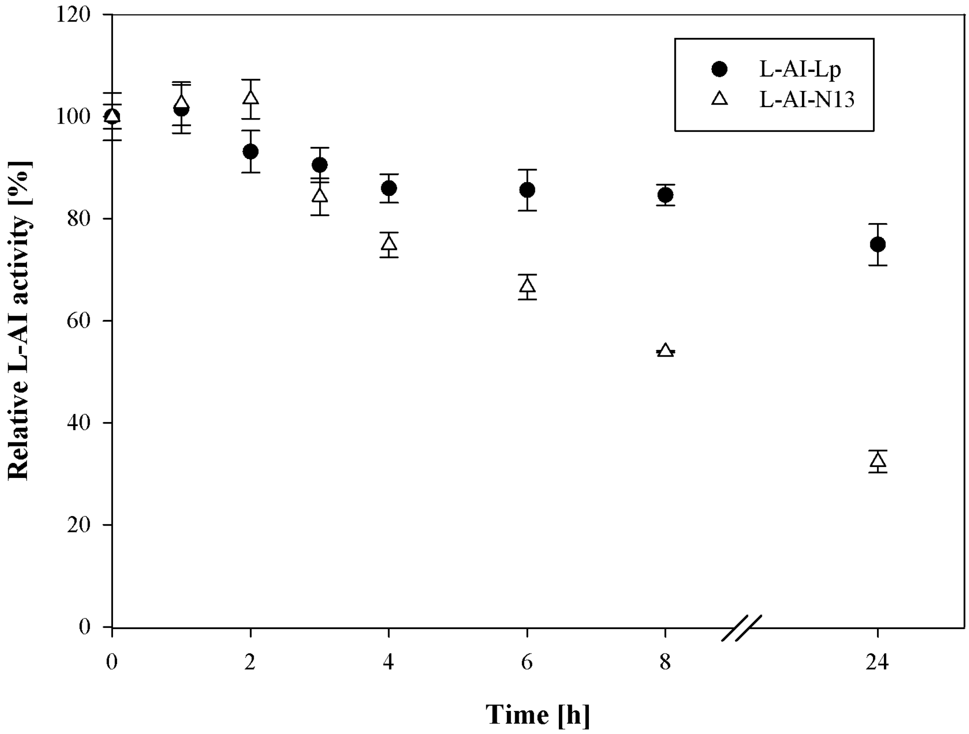

Figure S2. Thermostability of L-AI-Lp and L-AI-N13 in skim milk UF-permeate pH 6.5 at 60 °C. L-AI-Lp (1337.13 ± 62.06 $nkat_{D\text{-gal, 60 °C}}/mL_{enzyme\ solution}$) and L-AI-N13 (155.71 ± 3.68 $nkat_{D\text{-gal, 60 °C}}/mL_{enzyme\ solution}$) were incubated in skim milk UF-permeate and skim milk UF-permeate supplemented with 1 mM $MnCl_2$, respectively.

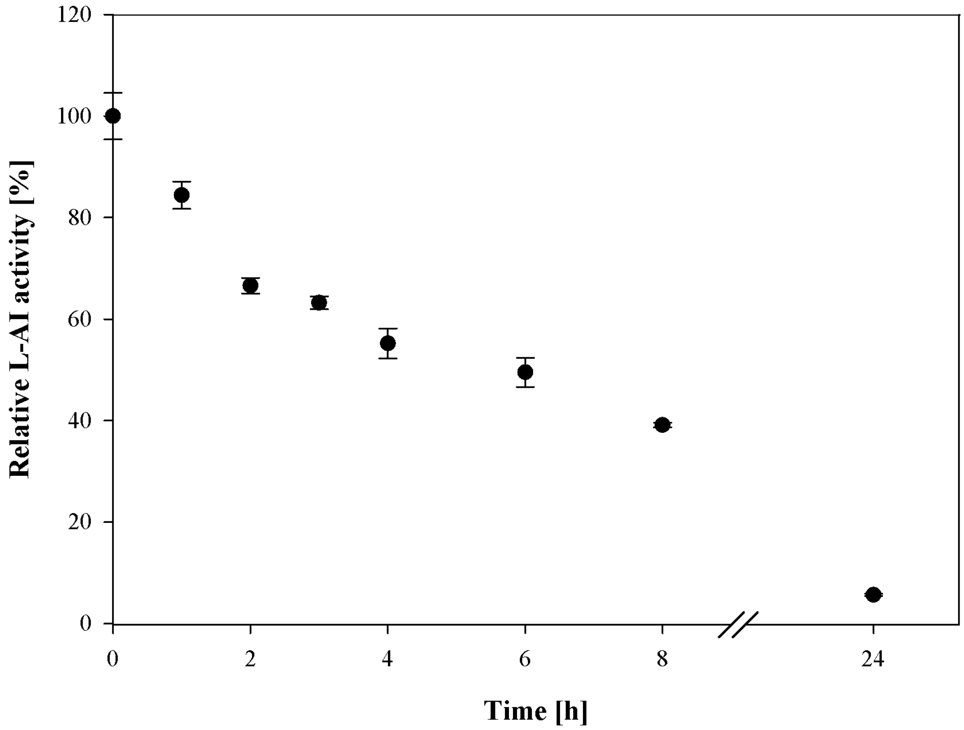

Figure S3. Thermostability of L-AI-Lp in skim milk UF-permeate pH 4.5 at 60 °C. L-AI-Lp (1123.06 ± 51.30 $nkat_{D\text{-gal, 60 °C}}/mL_{enzyme\ solution}$) was incubated in skim milk UF-permeate pH 4.5 supplemented with 1 mM $CoCl_2$.

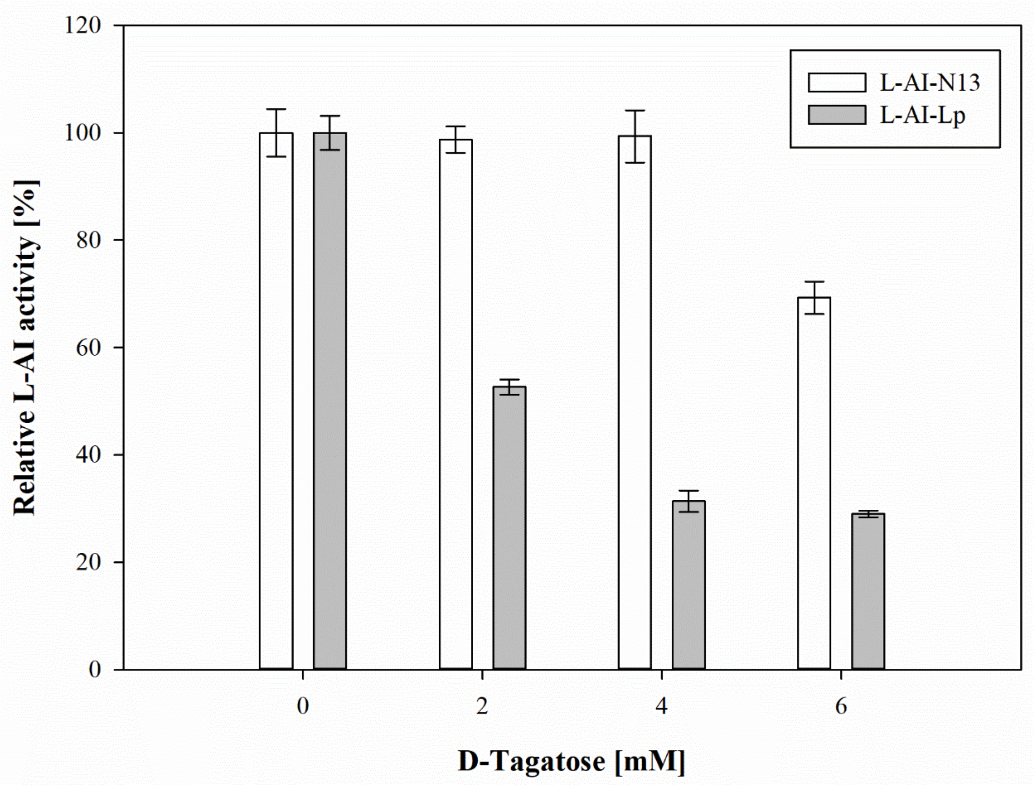

Figure S4. Influence of tagatose concentrations on the activity of L-AI-Lp and L-AI-N13. The activities of L-AI-Lp (100% = 996.35 ± 31.84 $nkat_{D\text{-gal, 60 °C}}/mL_{enzyme\ solution}$) and L-AI-N13 (100% = 418.57 ± 18.57 $nkat_{D\text{-gal, 60 °C}}/mL_{enzyme\ solution}$) were measured in skim milk UF-permeate and skim milk UF-permeate pH 6.5 supplemented with 1 mM $MnCl_2$, respectively.
